# Counter-Irritants

**Published:** 1842-09-03

**Authors:** 


*Counter-Irritants.*

The following formulæ have been communicated by Dr. Turnbull to the Editors of the Pharmaceutical Transactions.

*Tinctura Capsici Concentrati.*

℞. Capsici Baccarum, ℥iv.

Spiritus Vini Rect., ℥xij.

Macera per dies septem et cola. (It may also be made with advantage by displacement.)

This concentrated tincture is used as an external application, and is found to be a powerful rubefacient and counter-irritant, for which purpose the ordinary tincture of capsicum is not sufficiently potent.

Veratria, dissolved in this tincture, acquires increased activity; the capsicum apparently facilitating its absorption into the skin. Four grains of veratria, dissolved in an ounce of the concentrated tincture of capsicum, will be found as powerful in its effect as twelve or fifteen grains dissolved in alcohol.

*Pulvis Aluminis et Capsici.*

℞. Aluminis Sulphatis, partes tres.
Tinct. Capsici concentrati, partem unam.

Misce et sicca.

A very small quantity of this powder, applied to the tonsils, is found more efficacious, in some cases, than an alum and capsicum gargle.

*Unguentum Ipecacuanhæ.*

℞. Pulveris Ipecacuanhæ, ʒij.

Olei Olivæ, ʒij.

Adipis, ℥ss.

M. Ft. unguentum.

*Unguentum Emetinæ.*

℞. Emetinæ, g. xv.
Sp. Vini. Rect. q. s.
Adipis, ℥ss.
M. Ft. unguentum.

Dr. Turnbull states, that he has found this ointment particularly efficacious as a rubefacient in pulmonary and rheumatic affections, producing little or no pain or inconvenience to the patient.—*Medico-Chirurgical Review.* July, 1842.

